# Analysis of Visfatin Concentration and Other Potential Biomarkers Associated with MASLD Development in Saliva and Serum of Patients with Obesity—A Pilot Study

**DOI:** 10.3390/nu18040652

**Published:** 2026-02-16

**Authors:** Beata Zyśk, Joanna Smarkusz-Zarzecka, Urszula Cwalina, Agnieszka Gornowicz, Karolina Orywal, Anna Bielawska, Barbara Mroczko, Lucyna Ostrowska

**Affiliations:** 1 Department of Dietetics and Clinical Nutrition, Medical University of Bialystok, Mieszka I 4B Street, 15-054 Bialystok, Poland; joanna.smarkusz-zarzecka@umb.edu.pl (J.S.-Z.); lucyna.ostrowska@umb.edu.pl (L.O.); 2 Department of Biostatistics and Medical Informatics, Medical University of Bialystok, Szpitalna 37 Street, 15-295 Bialystok, Poland; urszula.cwalina@umb.edu.pl; 3 Department of Biotechnology, Medical University of Bialystok, Jana Kilinskiego Street 1, 15-089 Bialystok, Poland; agnieszka.gornowicz@umb.edu.pl (A.G.); anna.bielawska@umb.edu.pl (A.B.); 4 Department of Biochemical Diagnostics, Medical University of Bialystok, Waszyngtona 15A Street, 15-269 Bialystok, Poland; karolina.orywal@umb.edu.pl (K.O.); barbara.mroczko@umb.edu.pl (B.M.); 5 Department of Neurodegeneration Diagnostics, Medical University of Bialystok, Waszyngtona 15A Street, 15-269 Bialystok, Poland

**Keywords:** MASLD, obesity, biomarkers, visfatin, resistin, IL-6, IL-1β, MMP-2

## Abstract

**Background/Objectives**: Adipokines and cytokines, secreted by adipocytes and immune cells, play key roles in metabolic and inflammatory processes. This study aimed to assess the association between salivary visfatin levels and metabolic dysfunction-associated steatotic liver disease (MASLD), determine a salivary visfatin cutoff associated with increased risk of this disease, and examine correlations among selected adipokines, cytokines, and gelatinases in serum and saliva of obese patients. **Methods:** The study included 65 participants (40 women and 25 men) with a body mass index (BMI) ranging from 30.0 to 39.9 kg/m^2^, who were divided into groups based on whether the salivary visfatin concentration exceeded the quantification limit (1.229 ng/mL). Body composition analysis was performed using the bioelectrical impedance method, quantitative assessment of hepatic steatosis was carried out using transient elastography, and the concentrations of selected adipokines, cytokines, and gelatinases were determined in serum and saliva. **Results:** A relationship was observed between lower BMI and salivary visfatin concentrations below the quantification limit (*p* = 0.017), and between the absence of MASLD and visfatin levels below the quantification threshold in saliva (*p* = 0.05). Higher concentrations of interleukin-1β (*p* = 0.003) and matrix metalloproteinase-2 (*p* = 0.019) in saliva, as well as interleukin-6 (*p* = 0.002) in serum, were observed in the group with salivary visfatin levels above the quantification limit. Correlations were found between salivary and serum IL-6 concentrations (r = 0.30; *p* = 0.016) and between serum resistin and salivary IL-6 levels (r = 0.24; *p* = 0.056), as well as between serum IL-6 and salivary MMP-2 concentrations (r = 0.24; *p* = 0.059). **Conclusions:** In this pilot study, salivary visfatin levels were found to differ between obese individuals with and without MASLD and to be associated with selected anthropometric parameters and inflammatory markers, but the observed associations are exploratory and require confirmation.

## 1. Introduction

Adipose tissue acts as an endocrine organ and plays a key role in the development of metabolic complications linked to obesity. Through the secretion of adipokines and cytokines by adipocytes and immune cells, adipose tissue modulates both metabolic and inflammatory pathways that are critically involved in the pathogenesis of metabolic dysfunction-associated steatotic liver disease (MASLD) [1]. One of the adipokines secreted by adipose tissue—mainly by infiltrating macrophages—is visfatin, also known as nicotinamide phosphoribosyltransferase (NAMPT) or pre-B-cell colony-enhancing factor (PBEF). Available evidence from the literature indicates that visfatin may be associated with a range of metabolic disorders and disease states, including MASLD. However, the findings of studies conducted to date remain inconsistent and limited in number, and most of these studies were carried out on small populations [2,3,4]. Visfatin exists in the following two distinct forms: extracellular (eNAMPT) and intracellular (iNAMPT). In its extracellular form, the protein functions as an enzyme while also exhibiting cytokine-like activity—its concentration increases during inflammatory responses [1]. Given the pathophysiological mechanisms underlying MASLD, the assessment of visfatin levels in selected biological samples may represent a promising tool for identifying individuals at risk of developing or progressing MASLD.

Serum is the most commonly used biological material for the assessment of various biochemical parameters. However, saliva has received increasing attention in laboratory diagnostics because its collection is non-invasive, inexpensive, and does not require specialized equipment or trained medical personnel. Saliva is a secretion of the major and minor salivary glands and consists of 94–99% water, as well as numerous proteins, carbohydrates, and lipids, vitamins, cytokines, immunoglobulins, growth factors, and hormones. Given that saliva contains numerous biomarkers reflecting both metabolic and inflammatory status, it represents a promising diagnostic medium for systemic diseases [5,6,7]. Changes in salivary biomarker concentrations may reflect corresponding changes in blood levels. Compounds from the blood pass into saliva. Certain compounds are transferred between blood and saliva via passive diffusion or active transport; therefore, correlations may exist between their concentrations in saliva and serum [6,7]. Results from previous studies have shown that the concentrations of selected adipokines, including visfatin in saliva are measurable and differ between individuals with type 2 diabetes and those without diabetes, suggesting a potential diagnostic application of these salivary biomarkers [8,9]. However, the use of saliva for the assessment of adipokine and cytokine levels represents a novel approach in the diagnosis of MASLD.

The search for novel diagnostic methods and biomarkers represents an important direction in MASLD research, given the continuously increasing prevalence of the disease and the lack of effective tools for early diagnosis. According to the most recent epidemiological data, MASLD affects approximately 75.3% of individuals with obesity, with more than half of these patients (52.9%) already presenting with the more advanced form of the disease—metabolic dysfunction-associated steatohepatitis (MASH)—and significant hepatic fibrosis observed in 38.3% of cases. Notably, MASLD is also prevalent among individuals without obesity, affecting an estimated 18.3% of this population; among these patients, MASH and significant fibrosis are present in approximately 39.0% and 29.2% of cases, respectively. Despite this high disease burden across different phenotypes, early diagnosis of MASLD remains challenging due to nonspecific or absent clinical manifestations in the initial stages of the disease. Moreover, currently available diagnostic tools used in routine clinical practice are limited in their ability to reliably detect early-stage disease or to facilitate large-scale screening [10,11].

This study aimed to assess the association between salivary visfatin levels and MASLD, determine a salivary visfatin cutoff associated with increased risk of this disease, and examine correlations among selected adipokines, cytokines, and gelatinases in serum and saliva of obese patients.

## 2. Materials and Methods

### 2.1. Study Group

This observational cross-sectional study included 65 participants (40 women and 25 men) aged 22–55 years, selected by convenience sampling. The study enrolled individuals with class I obesity (BMI 30.0–34.9 kg/m^2^) and class II obesity (BMI 35.0–39.9 kg/m^2^) who provided a dental certificate confirming the absence of periodontal disease and oral inflammation. Individuals with a history of pharmacological or surgical obesity treatment, as well as those with eating disorders, were excluded from the study. Additional exclusion criteria included type 1 and type 2 diabetes mellitus, viral hepatitis, cholestatic or alcoholic liver disease, coexisting endocrine or hormonal disorders, pregnancy, lactation, and the presence of a cardiac pacemaker. The qualified participants were divided into two groups based on the quantification limit of salivary visfatin, established at 1.229 ng/mL. The study group (G1) consisted of 26 individuals with visfatin concentrations equal to or above the quantification threshold, while the control group (G0) included 39 individuals with visfatin concentrations below this limit.

Participants were recruited from individuals who voluntarily presented to the Department of Dietetics and Clinical Nutrition at the Medical University of Bialystok between July and August 2020. All participants received comprehensive information about the study objectives and procedures and were informed of their right to discontinue participation at any time. Written informed consent was obtained from each participant before inclusion in the study. The study protocols were approved by the Bioethics Committee of the Medical University of Bialystok (approval numbers R-I-002/647/2019, APK.002.468.2020, and APK.002.39.2021).

### 2.2. Nutritional Assessment

Participants underwent basic anthropometric measurements as well as body composition analysis. Body weight was measured to the nearest 0.01 kg, and height was measured to the nearest 0.5 cm, using a RADWAG WPT 100/200 OW scale with stadiometer (Radom, Poland). Waist and hip circumferences were measured with a standard anthropometric tape. Body mass index was calculated as body weight in kilograms divided by height in meters squared. The waist-to-hip ratio (WHR) was determined as the quotient of waist circumference to hip circumference, with both values expressed in centimeters.

Body composition was assessed using bioelectrical impedance analysis (BIA) with a BioScan 920-2 device (Essex, UK). Measurements were performed in the fasting state with participants in a supine position, using electrodes placed on the right upper and lower limbs. Total body fat percentage was evaluated. Additionally, the device allowed assessment of fat area, including visceral (cm^2^) and subcutaneous (cm^2^) fat. This analysis was performed with participants in a standing position, with electrodes positioned horizontally at the waist level. The results were processed using Maltron BioScan 920 v1.1 software, which enabled, among other parameters, the calculation of the visceral-to-subcutaneous fat ratio (VAT/SAT) [12].

Trained personnel carried out all anthropometric and body composition measurements following standardized procedures. Measurements were obtained using the same equipment throughout the study, which was operated in accordance with the manufacturer’s instructions and factory-calibrated settings. Prior to each measurement, the device functionality and electrode connections were checked to ensure proper performance.

### 2.3. Assessment of Liver Steatosis

**Figure 1:**
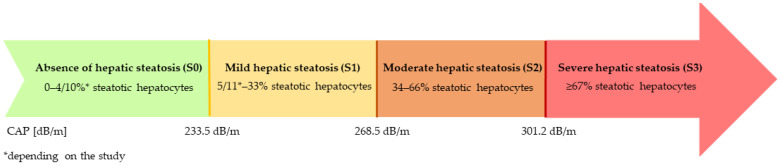
Adopted CAP thresholds for assessing the degree of hepatic steatosis.

Quantitative assessment of liver steatosis was performed using a FibroScan 530 Compact device (Echosens, Paris, France), which employs vibration-controlled transient elastography (VCTE). The measurement was enabled by the controlled attenuation parameter (CAP) function, which describes the attenuation of the ultrasound signal depending on the depth of penetration within the liver. Examinations were conducted in the fasting state with participants in a supine position and the right arm placed behind the head. M and XL probes were used depending on recommendations of manufacturer and routine clinical practice. The choice of probe was guided primarily by participants’ body mass index and abdominal adiposity, as well as by the ability to obtain reliable measurements with adequate signal quality and depth. Device was used with ultrasound gel applied on intercostal space. For each participant, at least 10 valid measurements were obtained. Measurement reliability was assessed using the interquartile range to median ratio (IQR/median), and only examinations with an IQR/median ≤ 30% were considered reliable and included in the final analysis [13]. Liver steatosis was defined based on the proportion of steatotic hepatocytes, according to the following thresholds:

Absence of hepatic steatosis (S0) → 0–4/10%* steatotic hepatocytes;
Mild hepatic steatosis (S1) → 5/11*–33% steatotic hepatocytes;
Moderate hepatic steatosis (S2) → 34–66% steatotic hepatocytes;
Severe hepatic steatosis → ≥67% steatotic hepatocytes.
* depending of the study
[14,15,16].
The CAP thresholds were taken from validated studies in adult NAFLD (non-alcoholic fatty liver disease) populations and were considered applicable to our cohort due to comparable metabolic and anthropometric characteristics. The adopted values presented in Figure 1.

### 2.4. Diagnosis of Metabolic Dysfunction-Associated Steatotic Liver Disease (MASLD)

To confirm that hepatic steatosis diagnosed in the participants was attributable to MASLD rather than another liver disease, the most recent diagnostic criteria were applied (in addition to prior exclusion of other liver conditions based on medical history).

MASLD was diagnosed in the presence of hepatic steatosis confirmed by imaging, with liver elastography applied in the present study, together with at least one metabolic risk factor. These included overweight or obesity (BMI ≥ 25 kg/m^2^ or waist circumference > 94 cm in men and >80 cm in women); impaired glucose metabolism, defined as fasting plasma glucose ≥ 5.6 mmol/L (100 mg/dL) or a 2 h plasma glucose value ≥ 7.8 mmol/L (140 mg/dL) during an oral glucose tolerance test; elevated blood pressure (≥130/85 mmHg) or ongoing antihypertensive therapy; hypertriglyceridemia (plasma triglycerides ≥ 1.70 mmol/L [150 mg/dL]) or lipid-lowering treatment; and reduced HDL-cholesterol concentrations (≤1.0 mmol/L [40 mg/dL] in men and ≤1.3 mmol/L [50 mg/dL] in women) or lipid-lowering therapy [17].

All participants with elastography-confirmed hepatic steatosis fulfilled the diagnostic criteria for MASLD. Among the 65 participants, MASLD was diagnosed in 54 individuals, whereas 11 participants had neither MASLD nor hepatic steatosis attributable to another disease entity.

Although participants were originally recruited based on the NAFLD definition, the updated MASLD nomenclature and diagnostic criteria were applied post hoc, following their publication, using data that had already been systematically collected at baseline. All metabolic criteria required for MASLD classification were assessed during the same study visit using standardized clinical and laboratory procedures. No additional measurements were performed for the purpose of reclassification.

### 2.5. Measurement of Selected Adipokines, Cytokines, Gelatinases and Biochemical Parameters in Serum

A fasting venous blood sample (10 mL) was collected from each participant in the morning (between 7:00 and 10:00 a.m.) at the same laboratory into a serum collection tube with a clot activator (S-Monovette, SARSTEDT, Numbrecht, Germany) and subsequently centrifuged. Blood serum was used for routine biochemical analyses, and the remaining serum samples were stored for the assessment of adipokine and cytokine concentrations. Fasting serum glucose, high-density lipoprotein cholesterol (HDL-C), and triglyceride levels (TG) were measured in the study participants using the ALINITY ci-series analyzer (Abbott, Wiesbaden, Germany). Serum concentrations of IL-6, IL-1β, resistin, MMP-2, and MMP-9 were quantified using commercially available Quantikine ELISA kits (R&D Systems, Minneapolis, MN, USA) according to the manufacturer’s instructions. Samples were aliquoted immediately after collection and stored at −80 °C until analysis, with only one freeze–thaw cycle before measurement.

### 2.6. Measurement of Selected Adipokines, Cytokines, and Gelatinases in Saliva

Prior to sample collection, participants rinsed their oral cavity with deionized water. Unstimulated saliva samples were then collected between 9:00 and 11:00 a.m. using the passive drool technique over approximately 15 min, yielding a total volume of about 5 mL per participant. All individuals were instructed to abstain from food and beverages, except water, for at least 2 h before saliva collection. The obtained saliva samples were homogenized and clarified by centrifugation at 1200 rpm for 15 min at 4 °C. The supernatant was aliquoted into smaller tubes and subsequently stored at −80 °C in order to avoid repeated freeze–thaw cycles; each aliquot was used for a single analytical determination. The analyses were performed immediately after sample collection. Concentrations of IL-6, IL-1β, resistin, MMP-2, and MMP-9 were measured by ELISA using high-sensitivity kits from R&D Systems Inc. (Minneapolis, MN, USA), whereas visfatin was determined using a high-sensitivity ELISA kit from Invitrogen (Thermo Fisher Scientific, Waltham, MA, USA), according to the manufacturers’ instructions [18].

### 2.7. Quality Control

Quality control parameters were defined for all analyzed biomarkers measured using ELISA assays. The minimum detectable dose (MDD), corresponding to the assay limit of detection (LOD), as well as intra-assay and inter-assay coefficients of variation (CVs), were reported to describe assay precision. Calibration procedures were based on manufacturer-provided standard curves and performed according to the manufacturers’ instructions. All assays were performed in duplicate.

All cytokines and adipokines, except visfatin, were quantifiable within the analytical ranges of the applied assays. The lower limit of quantification for salivary visfatin was 1.229 ng/mL, reflecting the analytical sensitivity of the assay. Salivary visfatin concentrations below this value could not be reliably detected or quantified.

Analytical performance characteristics, including minimum detectable dose (MDD), intra- and inter-assay coefficients of variation (CVs), and assay verification using the standard curves provided by the manufacturer, are summarized in the Supplementary Table S1.

### 2.8. Statistical Analysis

Statistical analyses were carried out using Statistica software (version 13 PL; TIBCO Software Inc., Palo Alto, CA, USA). The distribution of continuous variables was evaluated with the Shapiro–Wilk, Kolmogorov–Smirnov, and Lilliefors tests. As the assumption of normality was not met, non-parametric statistical methods were applied. Continuous data are presented as medians with interquartile ranges (25th–75th percentiles). Differences between two independent groups were analyzed using the Mann–Whitney U test, while relationships between categorical variables were assessed with the chi-square test of independence. Correlations between variables were examined using Spearman’s rank correlation analysis. The diagnostic value of salivary visfatin was evaluated by receiver operating characteristic (ROC) curve analysis, and the optimal cut-off value was determined using the Youden index. Sensitivity and specificity were calculated, and diagnostic accuracy was expressed as the area under the ROC curve (AUC) with corresponding 95% confidence intervals and *p*-values. Furthermore, multivariable logistic regression analysis was conducted to examine the association between salivary visfatin levels and MASLD after adjustment for potential confounding factors. Statistical significance was defined as a *p*-value below 0.05.

## 3. Results

A statistical analysis was performed for participants divided into groups based on the salivary visfatin quantification limit. The median age in group G1 was 47.5 years, while in group G0 it was 42.0 years; this difference was not statistically significant (*p* = 0.062). Group G1 consisted of 26 individuals—15 women (57.7%) and 11 men (42.3%). Group G0 included 39 individuals—25 women (64.1%) and 14 men (35.9%). There were no statistically significant differences between the groups with respect to sex (*p* = 0.611).

The characteristics of both groups, including anthropometric parameters, anthropometric indices, and body composition measures, are presented in Table 1.

Statistically significant differences between the groups were observed for BMI (*p* = 0.039) and VAT area at the waist level (*p* = 0.028). No statistically significant differences were found for the other analyzed parameters.

Table 2 presents the analysis of the relationship between salivary visfatin concentration, determined based on the quantification limit, and body mass index (BMI).

A statistically significant association was observed (*p* = 0.017) between less severe obesity (class I) and salivary visfatin concentrations below the quantification limit (1.229 ng/mL) for the device used. Among individuals with a class I obesity, 71.8% (*n* = 28) had visfatin concentrations below the quantification limit.

Table 3 presents the analysis of the relationship between salivary visfatin concentration, determined based on the quantification limit, and the presence of MASLD.

An association was observed between the absence of MASLD and salivary visfatin concentrations below the quantification limit (1.229 ng/mL), with a *p*-value of 0.05. Among individuals without MASLD, 90.9% (n = 10) had visfatin concentrations below the quantification limit. 

**Figure 2:**
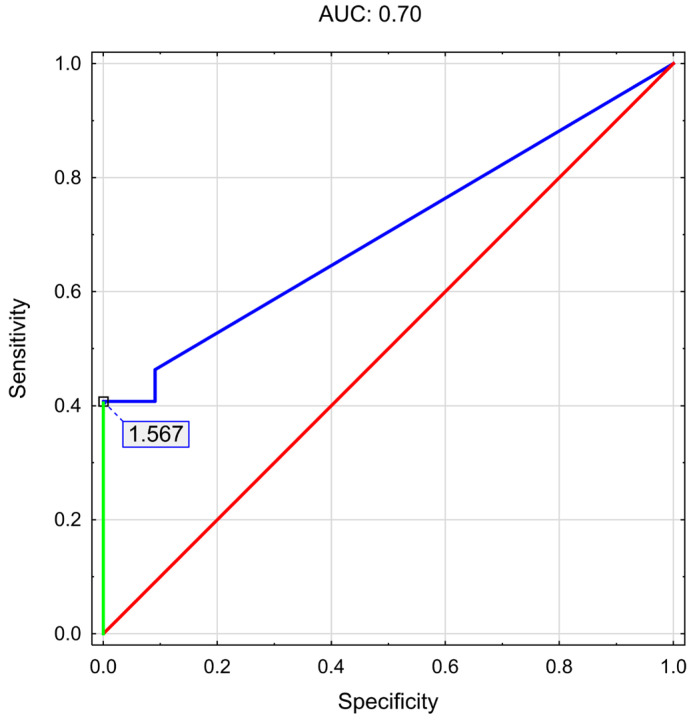
ROC curve analysis with the optimal cut-off determined using Youden’s index.

Subsequently, a ROC analysis was performed to determine the cut-off value of salivary visfatin associated with an increased risk of developing hepatic steatosis (assessed using the CAP parameter). The results are presented in Table 4 and Figure 2.

ROC analysis identified an optimal cut-off value for salivary visfatin of 1.567 ng/mL for hepatic steatosis (AUC: 0.70; *p* = 0.004; sensitivity: 41%; specificity: 100%). A multivariable logistic regression analysis was performed to assess the association between salivary visfatin concentrations (≥limit of quantification vs. <limit of quantification) and the presence of MASLD. The results are presented in Table 5.

An odds ratio (ORs) of 8.62 indicates that individuals with visfatin concentrations greater than or equal to the limit of quantification have 8.6-fold higher odds of hepatic steatosis compared with individuals whose visfatin levels were below the limit of quantification. OR is adjusted for gender, age, BMI, VAT/SAT, SBP, TG, and HDL.

Table 6 presents a comparison of salivary and serum concentrations of selected adipokines, cytokines and gelatinases between the groups.

Higher median salivary concentrations of IL-1β and MMP-2 were observed in group G1 compared with group G0, and these differences were statistically significant. A significantly higher median serum concentration of IL-6 was also found in group G1 than in group G0. No significant differences were observed for the other salivary or serum parameters between the groups.

A correlation analysis was performed between salivary and serum concentrations of adipokines, cytokines, and gelatinases in all participants (n = 65), and the results are presented in Table 7.

It was observed that only salivary IL-6 concentration showed a statistically significant moderate positive correlation with its serum concentration (r = 0.30; *p* = 0.016). Additionally, weak positive correlations approaching statistical significance were found between serum resistin and salivary IL-6 (r = 0.24; *p* = 0.056), as well as between serum IL-6 and salivary MMP-2 (r = 0.24; *p* = 0.059).

## 4. Discussion

### 4.1. Salivary Visfatin in MASLD: Associations and Diagnostic Potential

Studies conducted by other authors have investigated the relationship between visfatin and metabolic complications of obesity using serum or plasma as the biological matrix [19]. However, the present study is the first to employ saliva as a potential diagnostic material for assessing visfatin concentrations in individuals with MASLD and coexisting obesity. In the present study, significantly higher median BMI values (Me G1: 35.5 kg/m^2^ vs. G0: 33.5 kg/m^2^; *p* = 0.039), as well as a greater VAT area at the waist level (Me G1: 313.0 cm^2^ vs. G0: 257.0 cm^2^; *p* = 0.028), were observed in the group with higher salivary visfatin concentrations compared with the group in which visfatin levels were below the quantification limit. Moreover, a statistically significant association was identified (*p* = 0.017), indicating that individuals with less advanced obesity (lower BMI values) were more likely to present salivary visfatin concentrations below the quantification threshold. Conversely, in participants with more advanced obesity, visfatin concentrations more frequently exceeded the quantification limit. These findings are consistent with the systematic review and meta-analysis by Chang et al., which demonstrated that plasma visfatin concentrations were significantly elevated in individuals with overweight or obesity [20].

In the present study, approximately 91% of individuals without MASLD exhibited salivary visfatin concentrations below the quantification threshold. This pattern suggests an association between the absence of MASLD and lower salivary visfatin levels (*p* = 0.05). Due to recent revisions in terminology and diagnostic definitions, along with the scarcity of research focusing on MASLD, our results were primarily compared with studies conducted in NAFLD and MAFLD (metabolic dysfunction-associated fatty liver disease) populations. However, 99% of patients with NAFLD meet MASLD criteria [21]. Auguet et al. reported that plasma visfatin concentrations were higher in women with NAFLD compared with healthy controls [3]. In contrast, other authors did not demonstrate differences in visfatin concentrations between individuals with and without MAFLD, nor did they observe a significant association between serum visfatin levels and the development of this condition [22,23]. Moreover, the results of a systematic review and meta-analysis by Ismaiel et al. did not indicate an association between serum visfatin concentrations and the development of NAFLD or nonalcoholic steatohepatitis (NASH) [24]. Chen et al. updated this systematic review and obtained similar results in the general population of individuals with MAFLD. However, a subgroup analysis revealed that visfatin concentrations were significantly higher in patients with MAFLD and coexisting obesity compared with the control group [25]. These observations align with our findings. Based on the observed association, further analyses were conducted. The multivariable logistic regression analysis indicated that higher salivary visfatin concentrations (≥the limit of quantification) were associated with increased odds of MASLD, independent of potential confounders such as age, gender, BMI, VAT/SAT ratio, blood pressure, and lipid parameters, suggesting that visfatin levels may reflect obesity-related hepatic.

ROC analysis identified an optimal cut-off value for salivary visfatin (cut-off point 1.567 ng/mL); however, the obtained AUC (0.70) together with low sensitivity (41%) indicates that visfatin cannot be considered a standalone diagnostic marker of hepatic steatosis. Nevertheless, the very high specificity (100%) observed at this cut-off suggests a potential role of salivary visfatin as a rule-out biomarker. In individuals with salivary visfatin concentrations below the established threshold, hepatic steatosis was not observed, indicating that low visfatin levels may, with high probability, exclude the presence of hepatic steatosis. Therefore, salivary visfatin may be useful as a supportive pre-screening tool to identify individuals who are unlikely to require further, more invasive or costly diagnostic procedures, rather than as a marker for disease detection. The cut-off value was selected using the Youden index. Although alternative cut-off points were also evaluated, they did not result in a meaningful improvement in sensitivity, while leading to a reduction in specificity. The chosen threshold was characterized by the highest sensitivity achievable while maintaining 100% specificity. Given the exploratory nature of this analysis and the intended role of salivary visfatin as a supportive rather than standalone diagnostic marker, prioritizing specificity was considered methodologically justified. Nevertheless, this approach should be regarded as hypothesis-generating and requires validation in larger cohorts; therefore, the selected cut-off should be interpreted with caution, which is particularly important given that existing data suggest this study represents the first attempt to establish a predictive threshold for salivary visfatin in the context of MASLD development.

### 4.2. Visfatin-Related Inflammatory Pathways in MASLD

Visfatin exhibits pro-inflammatory properties by enhancing monocyte activity, inducing oxidative stress, activating nuclear factor kappa B (NF-κB), and promoting the production of cytokines, including IL-1β, TNF-α, and IL-6 [26]. Moreover, this adipokine enhances IL-6 gene expression in endothelial cells [27]. In studies by Heo et al., using the HepG2 cell line (an in vitro liver cell model), a significant increase in IL-6, TNF-α, and IL-1β concentrations was observed following exposure of HepG2 cells to visfatin [28]. It is well established that these pro-inflammatory cytokines play a key role in the development of insulin resistance and MASLD, as well as in the progression to MASH. They act as important mediators of inflammation and participate in multiple inflammatory pathways that disrupt insulin signaling, including the activation of intracellular kinases and the stimulation of C-reactive protein (CRP) production in the liver [29]. In the present study, participants with salivary visfatin concentrations above the quantification threshold exhibited significantly higher levels of IL-1β in saliva (Me G1: 844.2 pg/mL vs. G0: 331.2 pg/mL; *p* = 0.003) and IL-6 in serum (Me G1: 2.4 pg/mL vs. G0: 1.7 pg/mL; *p* = 0.002) compared with individuals whose salivary visfatin levels were below the quantification limit. Additionally, higher median salivary MMP-2 concentrations were observed in the group with elevated visfatin (Me G1: 1.2 ng/mL vs. G0: 0.8 ng/mL; *p* = 0.019). In studies by Heo et al., using cellular models, MMP-2 expression was shown to increase in response to factors secreted by macrophages and hepatic stellate cells following visfatin stimulation [28]. According to the available literature, alterations in gelatinase expression, including MMP-2, influence the fibrogenesis process by participating in the degradation of the extracellular matrix [30]. These findings thus support the notion that visfatin may initiate and accompany inflammatory–fibrotic activation in the course of MASLD.

### 4.3. Correlations Between Salivary and Serum Biomarkers

In the present study, additional correlations were analyzed between selected adipokines and cytokines in serum and saliva (IL-6, IL-1β, resistin, MMP-2, and MMP-9). A statistically significant, moderate, positive correlation was observed between IL-6 concentrations in serum and saliva (r = 0.30; *p* = 0.016). Consequently, salivary IL-6 may reflect systemic inflammatory activity in MASLD, particularly as it is a cytokine recognized as a mediator involved at every stage of MASLD pathogenesis: from the development of hepatic steatosis to progression to MASH and the development of hepatic fibrosis [31]. Other authors have also investigated the relationship between this cytokine in serum and saliva, although not in individuals with obesity, MASLD, or comorbid conditions. Parkin et al. reported a statistically significant, moderate, positive correlation between salivary and serum IL-6 concentrations (r = 0.465; *p* < 0.01) in older adults without dementia [32]. In contrast, Markos et al. reported a correlation between IL-6 concentrations in the assessed diagnostic materials (r = 0.465; *p* < 0.01) in individuals with inactive multiple sclerosis [33].

In the present study, serum IL-6 concentrations were positively correlated with salivary MMP-2 levels (r = 0.24; *p* = 0.059). This finding suggests that MMP-2 activity in saliva may reflect systemic metabolic inflammation, for which IL-6 acts as a key mediator. Both IL-6 and MMP-2 participate in distinct but interconnected signaling pathways that contribute to the development of hepatic fibrosis [34]. In our previous studies, correlations between salivary MMP-2 levels and the CAP (assessing liver steatosis via elastography) were analyzed in individuals without liver fibrosis or with minimal fibrosis. A statistically significant, positive correlation was observed (r = 0.37; *p* < 0.001) [35]. In the present study, a positive association was also observed between serum resistin concentrations and salivary IL-6 levels (r = 0.24; *p* = 0.056). Resistin is an adipokine that initiates a cascade of pro-inflammatory cytokines, including IL-6, which in turn stimulate hepatocytes to mount an inflammatory response [36]. The results suggest that activation of systemic, metabolically driven inflammatory processes by resistin may induce or enhance local IL-6 secretion in saliva. In their study, Mariadi et al. reported a strong, positive correlation between serum resistin and IL-6 concentrations in the same biological material (r = 0.51; *p* < 0.001) in individuals with liver cirrhosis [37]. Similarly, El-Shayeb et al. observed a very strong, positive correlation between the serum concentrations of these inflammatory mediators (r = 0.84; *p* < 0.001) in individuals with decompensated liver cirrhosis [38].

### 4.4. Limitations of the Study

This study has several limitations that should be acknowledged when interpreting the findings. Participant recruitment was conducted through mass media outlets, including regional radio and television stations as well as institutional social media channels. Individuals without access to these media platforms may not have been informed about the study, which could have introduced selection bias and limited the representativeness of the study population relative to the general population. The modest sample size could have reduced statistical sensitivity, making it more difficult to identify small effects and to generalize the results. Standardized effect size measures (e.g., Cliff’s delta) and confidence intervals were not calculated, as the primary aim of the analyses was hypothesis testing rather than estimation of non-parametric effect sizes. Finally, the lack of an external validation cohort limits the ability to confirm the robustness and reproducibility of the findings in independent populations. In light of the relatively small cohort and the absence of external validation, the ROC-derived cut-off value should be considered exploratory, with a potential risk of overfitting, and requires confirmation in larger, independent cohorts.

Body composition was assessed using bioelectrical impedance analysis (BIA), which is subject to several methodological constraints. BIA measurements may be influenced by hydration status, recent physical activity, and device-specific variability, potentially affecting the accuracy of body composition estimates. Nevertheless, standardized measurement conditions were applied to minimize these effects. Although liver biopsy remains the histological reference standard, steatosis was assessed using the controlled attenuation parameter (CAP), a widely recommended, validated, and non-invasive method in clinical practice; nevertheless, it represents an indirect assessment compared with histological evaluation. Saliva-based analyses also present inherent methodological challenges. The saliva collection method was not standardized for flow rate, which may have substantially influenced total protein content, biomarker concentrations, and osmolality.

## 5. Conclusions

In this pilot study, salivary visfatin levels were found to differ between obese individuals with and without MASLD and to be associated with selected anthropometric parameters and inflammatory markers, but the observed associations are exploratory and require confirmation. The results suggest that salivary assessment of visfatin and selected inflammatory markers represents a promising direction for future research. This will require well-powered longitudinal studies with standardized saliva collection protocols, external validation cohorts, and complementary mechanistic investigations.

## Figures and Tables

**Table 1:** Comparison of anthropometric parameters and body composition between the groups.

Parameter	G1 Visfatin Concentration ≥ LOQ		G0 Visfatin Concentration < LOQ		*p*
	Median	Q_1_–Q_3_	Median	Q_1_–Q_3_	
BMI (kg/m^2^)	35.5	32.6–38.0	33.5	31.6–35.3	0.039 *
Waist circumference (cm)	112.0	105.0–120.0	107.0	105.0–112.0	0.083
WHR	0.96	0.89–0.97	0.94	0.88–0.97	0.387
Total body fat content (%)	40.7	33.9–47.1	40.5	33.2–45.9	0.342
VAT (cm^2^)	313.0	220.0–350.0	257.0	156.0–350.0	0.028 *
SAT (cm^2^)	131.0	110.0–161.0	128.0	105.0–146.0	0.395
VAT/SAT	2.2	2.0–3.3	2.2	1.4–2.8	0.158

Values are presented as median and interquartile range (Q1–Q3); * *p*—statistical significance (*p* < 0.05); Abbreviations: LOQ—limit of quantification; BMI—body mass index; WHR—waist-to-hip ratio; VAT—visceral adipose tissue; SAT—subcutaneous adipose tissue; VAT/SAT ratio—ratio of visceral to subcutaneous adipose tissue.

**Table 2:** Relationship between salivary visfatin concentration and body mass index (BMI).

	Class II Obesity BMI = 35.0–39.9 kg/m^2^		Class I Obesity BMI = 30.0–34.9 kg/m^2^	
	*n*	%	*n*	%
Visfatin concentration < LOQ	11	42.3%	28	71.8%
Visfatin concentration ≥ LOQ	15	57.7%	11	28.2%
	26	100%	39	100%

Abbreviations: LOQ—limit of quantification (1.229 ng/mL)

**Table 3:** Relationship between salivary visfatin concentration and the presence of MASLD.

	MASLD		Absence of MASLD	
	*n*	%	*n*	%
Visfatin concentration < LOQ	29	53.7%	10	90.9%
Visfatin concentration ≥ LOQ	25	46.3%	1	9.1%
	54	100%	11	100%

Abbreviations: LOQ—limit of quantification (1.229 ng/mL).

**Table 4:** ROC analysis to determine the threshold salivary visfatin concentration associated with an increased risk of developing hepatic steatosis.

Parameter	Salivary Visfatin Concentration (ng/mL)
AUC (95% Cl)	0.70 (0.57–0.84)
*p*-Value AUC	0.004 *
Cut-off point	1.567
Sensitivity	41%
Specificity	100%

* *p*—statistical significance (*p* < 0.05); Abbreviations: AUC—area under the curve; Cl—confidence interval.

**Table 5:** Multivariable logistic regression analysis of the association between salivary visfatin concentrations and MASLD.

Adjusted for	OR	95% CI	*p*
-	8.62	(1.031, 72.114)	0.047 *
Gender	8.45	(1.007, 70.993)	0.049 *
Age	7.28	(0.853, 62.113)	0.070
BMI	6.56	(0.757, 56.930)	0.088
VAT/SAT	8.05	(0.950, 68.280)	0.056
SBP	8.50	(0.972, 74.217)	0.053
TG	9.52	(1.110, 81.663)	0.040 *
HDL	8.50	(1.008, 71.721)	0.049 *
All of the above	8.04	(0.748, 86.433)	0.085

* *p*—statistical significance (*p* < 0.05). Abbreviations: VAT/SAT ratio—ratio of visceral to subcutaneous adipose tissue; SBP—systolic blood pressure; TG—triglycerides; HDL—high-density lipoprotein cholesterol; Cl—confidence interval.

**Table 6:** Comparison of salivary and serum concentrations of selected adipokines, cytokines and gelatinases between the groups.

Parameter		G1 Visfatin Concentration ≥ LOQ		G0 Visfatin Concentration < LOQ		*p*
		Median	Q_1_–Q_3_	Median	Q_1_–Q_3_	
Saliva	IL-6 (pg/mL)	9.6	4.6–17.5	9.2	4.0–14.4	0.727
	IL-1β (pg/mL)	844.2	337.2–1418.3	331.2	217.9–611.2	0.003 *
	Resistin (ng/mL)	4.6	2.0–6.2	3.2	1.5–5.5	0.445
	MMP-2 (ng/mL)	1.2	0.9–1.8	0.8	0.7–1.2	0.019 *
	MMP-9 (ng/mL)	425.4	326.4–595.5	521.3	290.9–933.1	0.560
Serum	IL-6 (pg/mL)	2.4	1.9–2.9	1.7	1.2–2.1	0.002 *
	IL-1β (pg/mL)	0.5	0.0–1.0	0.3	0.0–0.7	0.471
	Resistin (ng/mL)	11.8	8.8–16.7	10.5	8.8–15.0	0.663
	MMP-2 (ng/mL)	351.0	270.5–441.1	331.5	270.8–480.2	0.763
	MMP-9 (ng/mL)	775.2	631.9–1210.2	884.7	657.6–1091.9	0.490

Values are presented as median and interquartile range (Q1–Q3); * *p*—statistical significance (*p* < 0.05). Abbreviations: LOQ—limit of quantification; IL-6—interleukin-6; IL-1β—interleukin-1β; MMP-2—matrix metalloproteinase-2; MMP-9—matrix metalloproteinase-9; ng/mL—nanograms per milliliter; pg/mL—picograms per milliliter.

**Table 7:** Correlations between salivary and serum concentrations of adipokines, cytokines, and gelatinases in all study participants.

Adipokines/Cytokines/Gelatinanes		IL-6 (pg/mL)	IL-1β (pg/mL)	Resistin (ng/mL)	MMP-2 (ng/mL)	MMP-9 (ng/mL)
	Salivary					
Serum						
IL-6 (pg/mL)		r = 0.30 *p* = 0.016 *	r = 0.21 *p* = 0.097	r = 0.06 *p* = 0.612	r = 0.24 *p* = 0.059	r = 0.22 *p* = 0.074
IL-1β (pg/mL)		r = 0.14 *p* = 0.282	r = 0.03 *p* = 0.829	r = 0.08 *p* = 0.552	r = 0.03 *p* = 0.841	r = −0.02 *p* = 0.894
Resistin (ng/mL)		r = 0.24 *p* = 0.056	r = −0.04 *p* = 0.768	r = 0.16 *p* = 0.198	r = 0.05 *p* = 0.712	r = 0.03 *p* = 0.794
MMP-2 (ng/mL)		r = −0.14 *p* = 0.282	r = 0.03 *p* = 0.818	r = 0.15 *p* = 0.225	r = 0.04 *p* = 0.724	r = 0.12 *p* = 0.337
MMP-9 (ng/mL)		r = −0.11 *p* = 0.392	r = 0.05 *p* = 0.685	r = −0.05 *p* = 0.664	r = −0.13 *p* = 0.321	r = 0.12 *p* = 0.351

* *p*—statistical significance (*p* < 0.05). Abbreviations: IL-6—interleukin-6; IL-1β—interleukin-1β; MMP-2—matrix metalloproteinase-2; MMP-9—matrix metalloproteinase-9; ng/mL—nanograms per milliliter; pg/mL—picograms per milliliter.

## Data Availability

The data presented in this study are available on request from the corresponding author. The data are not publicly available due to privacy and ethical reasons.

## Supplementary Materials

The following supporting information can be downloaded at https://www.mdpi.com/article/10.3390/nu18040652/s1, Table S1: Analytical performance characteristics of ELISA assays.

## Abbreviations

The following abbreviations are used in this manuscript:

AUC	Area under the curve
BIA	Bioelectrical impedance analysis
BMI	Body mass index
CAP	Controlled attenuation parameter
CI	Confidence interval
CRP	C-reactive protein
ELISAs	Enzyme-linked immunosorbent assays
eNAMPT	Extracellular nicotinamide phosphoribosyltransferase
G0	Control group
G1	Study group
HDL	High-density lipoprotein
IL-1β	Interleukin-1β
IL-6	Interleukin-6
iNAMPT	Intracellular nicotinamide phosphoribosyltransferase
LOQ	Limit of quantification
MAFLD	Metabolic dysfunction-associated fatty liver disease
MASH	Metabolic dysfunction-associated steatohepatitis
MASLD	Metabolic dysfunction-associated steatotic liver disease
MMP-2	Matrix metalloproteinase-2
MMP-9	Matrix metalloproteinase-9
NAFLD	Non-alcoholic fatty liver disease
NAMPT	Nicotinamide phosphoribosyltransferase
NASH	Nonalcoholic steatohepatitis
NF-κB	Nuclear factor kappa B
ng/mL	Nanograms per milliliter
PBEF	Pre-B-cell colony-enhancing factor
pg/mL	Picograms per milliliter.
ROC	Receiver Operating Characteristic
SAT	Subcutaneous adipose tissue
SBP	Systolic blood pressure
TG	Triglycerides
TNF-α	Tumor necrosis factor alpha
VAT	Visceral adipose tissue
VAT/SAT	Visceral-to-subcutaneous fat ratio
VCTE	Vibration-controlled transient elastography
WHR	Waist-to-hip ratio
