# Supplementary material for: Analysis of Visfatin Concentration and Other Potential Biomarkers Associated with MASLD Development in Saliva and Serum of Patients with Obesity—A Pilot Study
Source: Nutrients. 2026 Feb 16;18(4):652. doi: 10.3390/nu18040652 (PMC12943479; doi:10.3390/nu18040652)
Supplement: Supplementary file 1 [file nutrients-18-00652-s001.zip › nutrients-4048651-supplementary.pdf]

Table S1. Analytical performance characteristics of ELISA assays.

Parameter	IL-6	MMP-2	MMP-9	Resistin	IL-1β	Visfatin
Units	pg/mL	ng/mL	ng/mL	ng/mL	pg/mL	ng/ml
Assay type	ELISA	ELISA	ELISA	ELISA	ELISA	ELISA
Manufacturer	R&D Systems	R&D Systems	R&D Systems	R&D Systems	R&D Systems	Invitrogen/ Thermo Fisher Scientific
Catalog numbers	D6050 / S6050 / PD6050	MMP200 / SMMP200 / PMMP200	DMP900 / SMP900 / PDMP900	DRSN00 / SRN00 / PDRSN00	DLB50 / SLB50 / PDLB50	EH482RB
Minimum detectable dose (MDD)	0.70 pg/mL	0.033 ng/mL	0.156 ng/mL	0.026 ng/mL	1.0 pg/mL	1.1 ng/mL
Intra-assay CV (%)	1.6–4.4	3.6–7.0	2.0–2.9	3.8–5.3	3.3–8.5	<10
Inter-assay CV (%)	2.5–6.4	6.5–7.0	6.9–7.9	7.8–9.2	4.1–8.4	<12
Quality control samples	Low / Medium / High	Low / Medium / High	Low / Medium / High	Low / Medium / High	Low / Medium / High	Low / Medium / High
Acceptance criteria for QC	CV < 15%	CV < 15%	CV < 15%	CV < 15%	CV < 15%	CV < 12%
Sample matrix	Serum/ Saliva	Serum/ Saliva	Serum/ Saliva	Serum/ Saliva	Serum/ Saliva	Saliva
Storage temperature	−80 °C	−80 °C	−80 °C	−80 °C	−80 °C	−80 °C
Freeze–thaw cycles	1	1	1	1	1	1
Maximum storage duration	up to 24 months	up to 24 months	up to 24 months	up to 24 months	up to 24 months	up to 24 months

Concentrations of IL-6, IL-1β, resistin, MMP-2, and MMP-9 were measured in both serum and saliva using commercially available Quantikine ELISA kits (R&D Systems, Minneapolis, MN, USA), whereas visfatin was measured exclusively in saliva using a high-sensitivity ELISA kit from Invitrogen (Thermo Fisher Scientific, Waltham, MA, USA), according to the manufacturers' instructions. Limits of detection and intra- and inter-assay coefficients of variation were obtained from the manufacturers' datasheets. Serum samples were aliquoted immediately after collection, whereas saliva supernatants were aliquoted after centrifugation; all samples were thawed only once prior to analysis.
